# Trends in Survival Rates of Non–Small Cell Lung Cancer With Use of Molecular Testing and Targeted Therapy in Korea, 2010-2020

**DOI:** 10.1001/jamanetworkopen.2023.2002

**Published:** 2023-03-16

**Authors:** Sang Ah Chi, Hyeyeon Yu, Yoon-La Choi, Sehhoon Park, Jong-Mu Sun, Se-Hoon Lee, Jin Seok Ahn, Myung-Ju Ahn, Dae-Ho Choi, Kyunga Kim, Hyun Ae Jung, Keunchil Park

**Affiliations:** 1 Department of Health Sciences and Technology, Samsung Advanced Institute for Health Sciences and Technology, Sungkyunkwan University, Seoul, Republic of Korea; 2 Division of Hematology Oncology, Department of Medicine, Samsung Medical Center, Sungkyunkwan University School of Medicine, Seoul, Republic of Korea; 3 Department of Pathology and Translational Genomics, Samsung Medical Center, Sungkyunkwan University School of Medicine, Seoul, Republic of Korea; 4 Biomedical Statistics Center, Research Institute for Future Medicine, Samsung Medical Center, Seoul, Republic of Korea; 5 Department of Data Convergence and Future Medicine, Sungkyunkwan University School of Medicine, Seoul, Republic of Korea; 6 Department of Digital Health, Samsung Advanced Institute for Health Sciences and Technology, Sungkyunkwan University, Seoul, Republic of Korea

## Abstract

**Importance:**

Over the past 10 years, treatment of non–small cell lung cancer (NSCLC) has been continually revolutionized. However, standard clinical trials may not reflect current multiple lines of treatment and corresponding outcomes in a timely manner.

**Objective:**

To investigate outcomes associated with new treatment of NSCLC in a clinical setting.

**Design, Setting, and Participants:**

This cohort study included patients with NSCLC between January 1, 2010, and November 30, 2020, who received any anticancer treatment at Samsung Medical Center in Korea. Data were analyzed from November 2021 through February 2022.

**Exposures:**

Clinical and pathological stage, histology, and major druggable sequence variation, including epidermal growth factor receptor (EGFR), anaplastic lymphoma kinase (ALK), *ROS1*, *RET*, *MET* exon 14 skipping, *BRAF* V600E, *KRAS* G12C, and *NTRK* between 2 periods (period I: 2010-2015 vs period II: 2016–2020).

**Main Outcomes and Measures:**

The primary outcome was the 3-year survival rate of NSCLC. Secondary outcomes included median overall survival, progression-free survival, and recurrence-free survival.

**Results:**

Among 21 978 patients with NSCLC (median [range] age at diagnosis, 64.1 [57.0-71.0] years; 13 624 males [62.0%]), there were 10 110 patients in period I and 11 868 patients in period II; adenocarcinoma (AD) was the predominant histology (7112 patients [70.3%] in period I and 8813 patients [74.3%] in period II). There were 4224 never smokers [41.8%] in period I and 5292 never smokers [44.6%] in period II. Compared with patients in period I, patients during period II were more likely to undergo molecular tests in the AD (5678 patients [79.8%] vs 8631 patients [97.9%]) and non-AD (1612 of 2998 patients [53.8%] and 2719 of 3055 patients [89.0%]) groups. In patients with AD in period I, 3-year survival rates were 92.8% (95% CI, 91.8%-93.7%), 72.4% (95% CI, 68.3%-76.8%), 56.7% (95% CI, 53.4%-60.2%), and 28.7% (95% CI, 27.0%-30.4%) for stage I, II, III, and IV, respectively. In period II, 3-year survival rates of patients with AD were 95.1% (95% CI, 94.4%-95.9%), 82.5% (95% CI, 79.1%-86.1%), 65.1% (95% CI, 61.8%-68.6%), and 42.4% (95% CI, 40.3%-44.7%) for each stage, respectively. In patients without AD, 3-year survival rates were 72.0% (95% CI, 68.8%-75.3%), 60.0% (95% CI, 56.2%-64.1%), 38.9% (95% CI, 35.6%-42.5%), and 9.7% (95% CI, 7.9%-12.1%) for each stage in period I. In period II, the 3-year survival rates of patients without AD were 79.3% (95% CI, 76.3%-82.4%), 67.3% (95% CI, 62.8%-72.1%), 48.2% (95% CI, 44.5%-52.3%), and 18.1% (95% CI, 15.1%-21.6%) for each stage.

**Conclusions and Relevance:**

In this cohort study of 10 years of clinical data, survival outcomes were improved across all stages, with larger increases in patients with stage III to IV disease. The incidence of never-smokers and the use of molecular testing increased.

## Key Points

**Question:**

Was anticancer treatment for patients with non–small cell lung cancer (NSCLC) improved over a recent 10-year period in clinical practice?

**Findings:**

In this cohort study of 21 978 patients with NSCLC, 3-year survival rates in 2016 to 2020 were significantly higher than in 2010 to 2015 across all stages, with larger differences in patients with stage III to IV disease. The proportion of never smokers increased in 2016 to 2020 compared with 2010 to 2015, and patients in 2016 to 2020 were more likely to undergo molecular tests.

**Meaning:**

The results of this large-scale cohort study may reflect changes in survival outcomes for patients with NSCLC in a timely manner.

## Teaser

This cohort study investigates survival rates for non–small cell lung cancer in Korea from 2010 to 2020 with use of molecular testing and targeted therapy.

## Introduction

Lung cancer is the leading cause of cancer-related death worldwide.^1,2^ Over the past decade, there has been a major paradigm shift in the diagnosis and treatment of non–small cell lung cancer (NSCLC). In the early 2000s, the median overall survival of patients with recurrent or metastatic NSCLC was 14 to 16 months with platinum-based chemotherapy.^3^ Since the development of gefitinib,^4^ various types of targeted therapies have been developed, and the era of personalized medicine has begun. Oncogene-addicted NSCLC, which is characterized by the presence of epidermal growth factor receptor (EGFR), anaplastic lymphoma kinase (ALK), and *ROS1* was well known. In the middle of the 2010s, a clinical trial with next-generation EGFR-tyrosine kinase inhibitors (TKIs) and immunotherapy, including programmed death-1 (PD-1) and programmed death-ligand 1 (PD-L1) inhibitors, was started. The complexity of the treatment landscape for advanced NSCLC was further complicated by the advent of next-generation TKIs and immune checkpoint inhibitors and stratification of therapies by PD-L1 status.^5,6^ In addition, new but rare sequence variations, such as *RET* rearrangement, the *BRAF* V600E variant, *MET* exon 14 skipping, *KRAS* G12C variant, and *NTRK1/2/3* gene fusion, were found, and targeted therapy for these variants was approved by the Korean Food and Drug Administration (KFDA). Recently, new emerging variants, such as human epidermal growth factor receptor 2 (ERBB2) and *NRG* fusion, and new drug mechanisms, such as antibody-drug conjugates, have been studied.^7,8,9^

This area of research has progressed daily, with discoveries of new driver variants and development of targeted therapy and combination treatment strategies. As a result of these efforts, the journey of patients with NSCLC has become diverse and involves multiple processes, and the overall treatment duration has been extended. Targeted therapy or immunotherapy yields higher response rates, longer progression-free survival (PFS) and overall survival (OS), and a tolerable safety profile compared with cytotoxic chemotherapy; therefore, combination therapy with other drugs and sequential treatment could be possible after treatment failure. The classical model of randomized clinical trials may not reflect and analyze current multiple lines of treatment and their outcomes in a timely manner. This study investigated the trend of molecular diagnosis and advanced targeted therapy with better treatment outcomes of NSCLC in a clinical setting over a recent 10-year period.

## Methods

This cohort study follows the Strengthening the Reporting of Observational Studies in Epidemiology (STROBE) reporting guideline for observational studies. The study was reviewed and approved by the Institutional Review Board (IRB) of the Samsung Medical Center. The need for informed consent was waived by the IRB owing to the retrospective nature of this study.

### Study Participants and Data Collection

Patients who were diagnosed with histologically confirmed stage I to IV NSCLC and received anticancer treatment (eg, surgery, systemic treatment, radiotherapy, and gamma knife radiosurgery) at Samsung Medical Center between January 1, 2010, and November 30, 2020, were included. We used an algorithm developed in house to retrieve a medical database cohort called Real-time Automatically Updated Data Warehouse in Healthcare (ROOT-HEALTH).^10^

### Definition of Variables

Clinical stage was defined according to the American Joint Committee on Cancer. Patient demographic characteristics, including age, sex, smoking history, performance status, and pathological findings (such as major histology, histological subtype, cellular differentiation, primary tumor size, vascular invasion, lymphatic invasion, perineural invasion, extracapsular extension, and pleural invasion), were reviewed. For biomarker tests, immunohistochemistry (IHC), fluorescent in situ hybridization, real-time polymerase chain reaction (PCR), droplet digital PCR, and next-generation sequencing (NGS) were performed in a timely manner.

EGFR variation–positive NSCLC was identified using a peptide nucleic acid clamp (PNAclamp kit; Panagene), Cobas EGFR Mutation Test version 2 (Roche Diagnostics), droplet digital PCR, or NGS (CancerSCAN [Geninus], TruSight Oncology 500 [TSO 500; Illumina], Oncomine (ThermoFisher), and Guardant360 [Guardant Health]). Patients with common EGFR variants (deletion exon 19 or EGFR exon 21 p.L858R), uncommon EGFR variants (G719X, L861Q, S790I, E709K, L747S, H835Y, or de novo T790M), and EGFR exon 20 insertions were categorized as having EGFR variation–positive NSCLC. ALK-positive NSCLC was identified by ALK (D5F3) IHC or fluorescent in situ hybridization. *ROS1* rearrangement, *RET* rearrangement, *BRAF* V600E variation, *MET* exon 14 skipping, *KRAS* G12C variant, and *NTRK1/2/3* gene fusion positivity were identified using NGS. PD-L1 expression was evaluated with various IHC platforms using 22C3 antibody or SP 263 antibody and assessed using the tumor proportion score, the proportion of PD-L1 positive tumor cells out of 100 tumor cells. Subgroups of PD-L1 expression were classified as high expressor (PD-L1 ≥50%), expressor (PD-L1 ≥1% and <50%), and nonexpressor (PD-L1 <1%).

### Outcomes

The primary outcome was a 3-year survival rate from the first date of NSCLC diagnosis in period I (2010-2015) and period II (2016-2020). Secondary outcomes included OS, PFS, and recurrence-free survival (RFS).

### Statistical Analysis

For patient characteristics, all categorical variables are presented as numbers and percentages. We used χ^2^ and Fisher exact tests to compare general characteristics between period I and period II in the adenocarcinoma (AD) group (ie, patients with AD or adenosquamous carcinoma) and non-AD group (ie, patients with squamous cell carcinoma, neuroendocrine carcinoma, or unspecific NSCLC). The standardized mean difference (SMD) was calculated to compare the distribution of variables between groups.

The all-data cutoff date for analyses was November 30, 2021. Participants were described as censored if they did not recur or progress until November 30, 2021, or if they were dropped out from the cohort due to death. All statistical analyses were performed using R statistical software version 3.6.1 (R Project for Statistical Computing). Statistical significance was set at a 2-sided *P* value <.05. Data were analyzed from November 2021 and February 2022.

## Results

### Patient Characteristics

Among 21 978 patients who were diagnosed with NSCLC between January 2010 and November 2020 (median [range] age at diagnosis, 64.1 [57.0-71.0] years; 13 624 males [62.0%]), there were 10 110 patients in period I and 11 868 patients in period II. AD was the predominant histology (15 925 patients [72.5%]) with AD vs 6053 patients [27.5%] without AD; 7112 patients (70.3%) in period I vs 8813 patients (74.3%) in period II had AD, while 2998 patients (29.7%) in period I vs 3055 patients (25.7%) in period II did not have AD (SMD = 0.09; *P* < .001) (Table). Regardless of histologic type, 4224 patients [41.8%] and 5292 patients [44.6%] were never smokers in period I and period II (SMD = 0.06; *P* < .001).

**Table.** Patient Characteristics by Histology and Period

Characteristic	Overall, No. (%) (N = 21 978)	AD group (n = 15 925)				Non-AD group (n = 6053)			
		No. (%)		SMD	*P* value^c^	No. (%)		SMD	*P* value^c^
		Period I (n = 7112)^a^	Period II (n = 8813)^b^			Period I (n = 2998)	Period II (n = 3055)		
Age at diagnosis, median (range), y	64.1 (57.0-71.0)	61.2 (54.3-68.9)	63.4 (56.8-70.4)	0.19	<.001	67.2 (60.5-72.7)	68.1 (61.8-74.2)	0.14	<.001
Sex									
Male	13 624 (62.0)	3659 (51.4)	4519 (51.3)	<0.001	.84	2689 (89.7)	2757 (90.2)	0.02	.50
Female	8354 (38.0)	3453 (48.6)	4294 (48.7)			309 (10.3)	298 (9.8)		
ECOG performance status^d^									
0	7935 (36.1)	1848 (26.0)	4534 (51.4)	0.49	<.001	415 (13.8)	1138 (37.3)	0.54	<.001
1	10 531 (47.9)	3526 (49.6)	3568 (40.5)			1782 (59.4)	1655 (54.2)		
2-3	1081 (4.9)	408 (5.7)	205 (2.3)			318 (10.6)	150 (4.9)		
Smoking history^d^									
Never	9516 (43.3)	3882 (54.6)	4887 (55.5)	0.02	.26	342 (11.4)	405 (13.3)	0.06	.04
Former or current	12 413 (56.5)	3221 (45.3)	3909 (44.4)			2639 (88.0)	2644 (86.5)		
Histology									
AD	15 863 (72.2)	7079 (99.5)	8784 (99.7)	0.02	.22	0	0	0.03	.48
Adenosquamous carcinoma	62 (0.3)	33 (0.5)	29 (0.3)			0	0		
Squamous cell carcinoma	4870 (22.2)	0	0			2409 (80.4)	2461 (80.6)		
Neuroendocrine carcinoma	258 (1.2)	0	0			137 (4.6)	121 (4.0)		
NSCLC, unspecified	925 (4.2)	0	0			452 (15.1)	473 (15.5)		
BMI^d^^,^^e^									
Underweight	803 (3.7)	233 (3.3)	258 (2.9)	0.05	.03	163 (5.4)	149 (4.9)	0.07	.07
Normal	8119 (36.9)	2684 (37.7)	3190 (36.2)			1124 (37.5)	1121 (36.7)		
Overweight	5778 (26.3)	1903 (26.8)	2344 (26.6)			785 (26.2)	746 (24.4)		
Obesity	6967 (31.7)	2220 (31.2)	2931 (33.3)			857 (28.6)	959 (31.4)		
Clinical stage^d^									
I	9099 (41.4)	2972 (41.8)	4476 (50.8)	0.18	<.001	769 (25.7)	882 (28.9)	0.04	<.001
II	2217 (10.1)	443 (6.2)	617 (7.0)			619 (20.6)	538 (17.6)		
III	3412 (15.5)	819 (11.5)	949 (10.8)			788 (6.3)	856 (28.0)		
IV	6944 (31.6)	2674 (37.6)	2724 (30.9)			790 (26.4)	756 (24.7)		

Abbreviations: AD, adenocarcinoma; BMI, body mass index (calculated as weight in kilograms divided by height in meters squared); ECOG, Eastern Cooperative Oncology Group; NSCLC, non–small cell lung cancer; SMD, standardized mean difference.

^a^ 2010 to 2015.

^b^ 2016 to 2020.

^c^ χ^2^ test, Fisher exact test (for categorical variables), or 2-sample *t* test (for continuous variables) were used for the distribution of characteristics between periods.

^d^ There were missing values in the data: 2431 patients (11.1%), 49 patients (0.2%), 311 patients (1.4%), and 306 patients (1.4%) of the entire population for ECOG performance status, smoking history, BMI, and clinical stage, respectively.

^e^ BMI was classified as normal (18.5-22.9), underweight (<18.5), overweight (23.0-24.9), and obesity (≥25).

**Figure 1.**
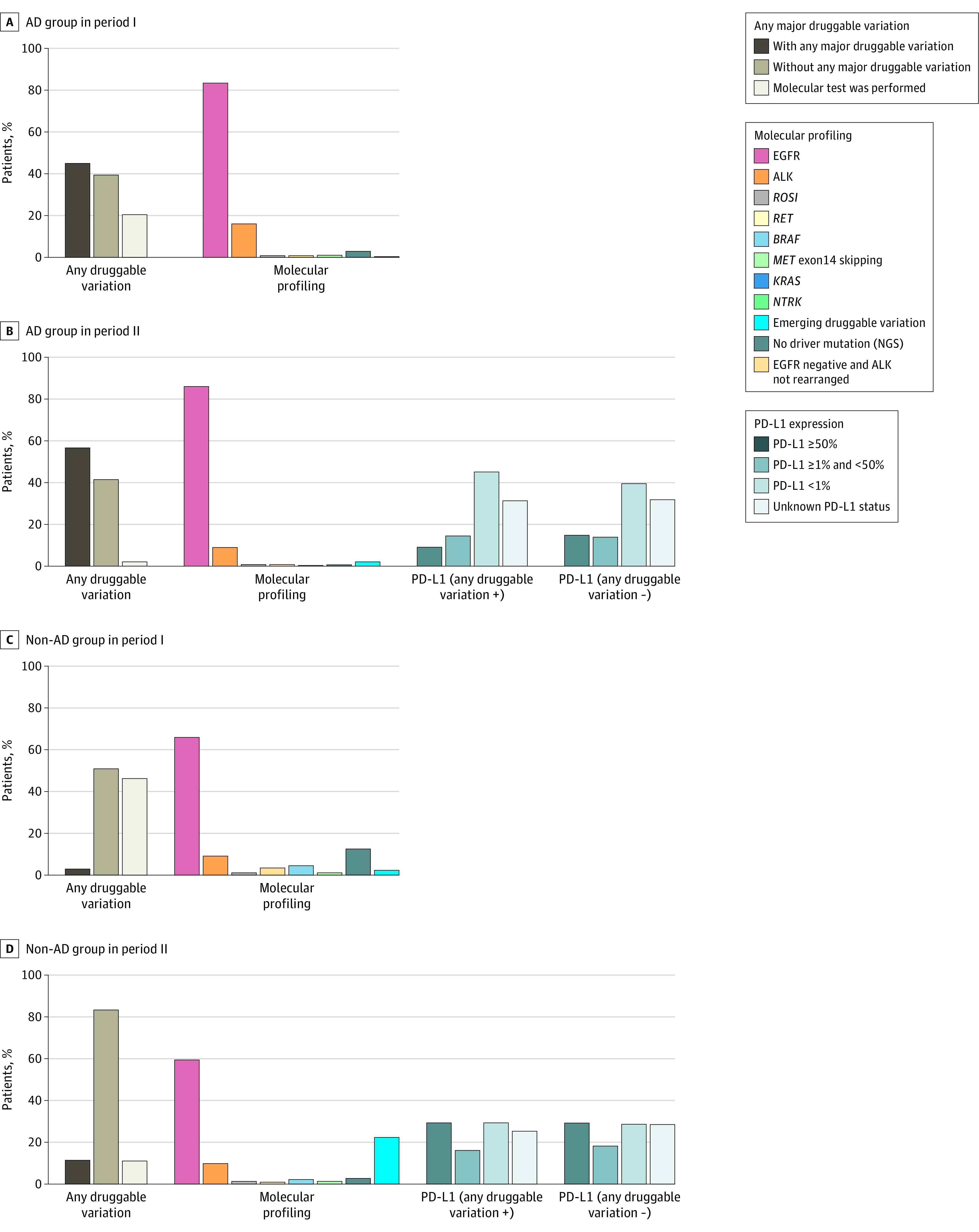
Molecular Profiling by Histology and Period Patients with any major druggable sequence variation included those with epidermal growth factor receptor (EGFR) variation, anaplastic lymphoma kinase (ALK) rearrangement, *ROS1* rearrangement, *RET* rearrangement, *BRAF* V600E variation, *MET* exon 14 skipping, *KRAS* G12C variation, or *NTRK* 1/2/3 gene fusion. Patients without any major druggable variation included those with emerging druggable variations (*NRAS*, *HRAS*, human epidermal growth factor receptor 2 [ERBB2], *PIK3CA*, *FGFR*, *RAF1*, and *TSC*), no driver variations, or EGFR and ALK wild type or those who did not undergo molecular tests. For AD groups, subtypes of EGFR positive are additionally illustrated. NGS indicates next-generation sequencing; PD-L1, programmed death-ligand 1.

At the time of initial diagnosis, 9099 patients [41.4%], 2217 patients [10.1%], 3412 patients [15.5%], and 6944 patients [31.6%] were in clinical stages I, II, III, and IV, respectively. In period I, 3741 patients [37.0%], 1062 patients [10.5%], 1607 patients [15.9%], and 3464 patients [34.3%] were in clinical stages I, II, III, and IV, respectively. In period II, 5358 patients [45.1%], 1155 patients [9.7%], 1805 patients [15.2%], and 3480 patients [29.3%] were in each stage, respectively (SMD = 0.16; *P* < .001). In period II, clinical stage I to II was predominant compared with period I, especially in the AD group (eFigure 1 in Supplement 1). For each year, 343 of 855 patients (40.1%), 419 of 954 patients (43.9%), 533 of 1063 patients (50.1%), 653 of 1337 patients (48.9%), 780 of 1469 patients (53.1%), and 687 of 1230 patients (55.8%) in period I and 882 of 1575 patients (56.0%), 997 of 1809 patients (55.2%), 1016 of 1788 patients (56.8%), 1085 of 1758 patients (61.7%), and 1113 of 1836 patients (60.6%) in period II had clinical stage I to II. Molecular profiles of AD and non-AD groups by period are shown in Figure 1 and eTable 1 in Supplement 1. In period I, 2877 patients (40.5%) in the AD group had at least 1 major druggable sequence variation: EGFR variation (2410 patients), ALK rearrangement (306 patients), *ROS1* rearrangement (24 patients), *RET* rearrangement (23 patients), *BRAF* V600E variation (28 patients), *KRAS* G12C variation (85 patients), and *NTRK1/2/3* gene fusion (1 patient). In period II, 4985 patients (56.6%) in the AD group had at least 1 major druggable sequence variation from more active molecular testing than period I (SMD = 0.67): EGFR variation (4376 patients), ALK rearrangement (457 patients), *ROS1* rearrangement (40 patients), *RET* rearrangement (41 patients), *BRAF* V600E variation (22 patients), *MET* exon 14 skipping (14 patients), *KRAS* G12C variation (34 patients), and *NTRK1/2/3* gene fusion (1 patient). Among patients without AD, 86 patients [2.9%] and 174 patients [5.7%] had any major druggable sequence variation in periods I and II, respectively. In the non-AD group, there was a large difference in the proportion of patients who underwent any molecular tests in period I vs period II (1612 patients [53.8%] vs 2719 patients [89.0%]; SMD = 0.86); there was also a difference in the AD group (5678 patients [79.8%] vs 8631 patients [97.9%]; SMD = .60). In 6786 patients with EGFR variation–positive NSCLC in the AD group, 3525 patients [51.9%], 380 patients [5.6%], 520 patients [7.7%], and 2327 patients [34.3%] were clinical stages I, II, III, and IV, respectively. In 763 patients with ALK-positive AD, 218 patients [28.6%], 44 patients [5.8%], 126 patients [16.5%], and 369 patients [48.5%] were clinical stages I, II, III, and IV, respectively. For subtypes of EGFR variation, 2410 patients with EGFR variations in the AD group had the following variations: exon 19 deletion (1235 patients), L858R (1016 patients), G719X (78 patients), insertion 20 (35 patients), L861Q (17 patients), S768I (4 patients), and de novo T790M (15 patients) in period I. In period II, 4376 patients with EGFR variations in the AD group had the following variations: exon 19 deletion (2118 patients), L858R (1837 patients), G719X (140 patients), insertion 20 (155 patients), L861Q (58 patients), S768I (7 patients), de novo T790M (19 patients), and E709K (1 patient).

### Treatment Patterns and Outcomes in Patients With Clinical Stages I-III NSCLC

Among patients with AD, 7288 of 7448 patients with stage I [97.9%], 974 of 1060 patients with stage II [91.9%], and 284 of 1061 patients with stage IIIA [26.8%] NSCLC received curative intent surgery as the first anticancer treatment. While the interaction effect of the pathological stage and 3 groups according to EGFR and ALK variation (EGFR variation–positive, ALK-positive, or EGFR and ALK wild type NSCLC) was significant, pathological stage was associated with risk of recurrence (adjusted hazard ratio, 3.92; 95% CI, 3.17-4.84 for stage II vs I; 6.07; 95% CI, 4.86-7.59 for stage IIIA vs I; all *P* < .001) (eFigure 2 in Supplement 1). The difference in recurrence of NSCLC was significant by pathological stage in each sequence variation type in the AD group (eFigure 2 in Supplement 1). Among patients with pathological stage IIIA who underwent curative intent surgery, 339 patients with EGFR variation–positive and 60 patients with ALK-positive NSCLC had inferior RFS than 297 patients with EGFR and ALK wild type NSCLC (median RFS was 25.2 months [95% CI, 22.6 to 30.6 months], 48.8 months [95% CI, 31.2 to 107.4 months], and 53.1 months [95% CI, 40.2 to 81.5 months] in patients with AD and EGFR variation–positive, ALK-positive, and EGFR and ALK wild type NSCLC, respectively) (eFigure 3 in Supplement 1).

Initial and sequential treatment patterns in patients with clinical stages IIIA and IIIB/C are shown in eFigure4 in Supplement 1. In 2060 patients with clinical stage IIIA, 573 patients (27.8%) received up-front surgery or neoadjuvant concurrent chemoradiotherapy followed by surgery. Among 1351 patients with clinical stage IIIB/C, 879 patients (65.1%) received definitive concurrent chemoradiotherapy.

### Treatment Patterns and Progression-Free Survival in Patients With Recurrent or Metastatic NSCLC

In this study, 4723 patients had recurrent or metastatic NSCLC in period I, and 4782 patients had recurrent or metastatic NSCLC in period II. In patients with EGFR variation–positive AD, the median PFS of first-line EGFR-TKI was 16.6 months (95% CI, 16.0 to 17.7 months) and 17.4 months (95% CI, 16.7 to 18.4 months) in period I and II, respectively.

The median PFS of first line ALK-TKI was 20.3 months (95% CI, 14.5 to 31.4 months) and 25.4 months (95% CI, 19.4 to 34.3 months) in periods I and II, respectively. The median OS was 91.5 months (95% CI, 74.5 to 114.5 months) in patients with advanced-stage, ALK-positive NSCLC in period I.

In patients with AD and EGFR and ALK wild type NSCLC, 1093 of 1337 patients (81.8%) and 1027 of 1272 patients (80.7%) received platinum-based chemotherapy during periods I and II, respectively. In period II, 152 patients [12.0%] received PD-1/PD-L1 inhibitors alone or in combination with chemotherapy. In period I, the median PFS was 5.5 months (95% CI, 5.2 to 5.8 months) and 8.8 months (95% CI, 4.0 to 76.3 months) for platinum-based chemotherapy and PD-1/PD-L1 inhibitor in combination with chemotherapy, respectively. Moreover, in period II, the median PFS was 5.2 months (95% CI, 5.0 to 5.4 months) and 8.0 months (95% CI, 6.3 to 10.2 months) for platinum-based chemotherapy and PD-1/PD-L1 inhibitor in combination with chemotherapy, respectively.

In patients without AD, 477 of 547 patients (87.2%) and 644 of 826 patients (78.0%) received platinum-based chemotherapy during periods I and II, respectively. In period II, 129 patients (15.6%) received PD-1/PD-L1 inhibitors alone or in combination with chemotherapy. In period I, the median PFS was 5.0 months (95% CI, 4.7 to 5.4 months) and 2.3 months (95% CI, 2.1 to 6.8 months) for platinum-based chemotherapy and PD-1/PD-L1 inhibitor in combination with chemotherapy, respectively. In period II, the median PFS was 3.9 months (95% CI, 3.5 to 4.2 months) and 4.6 months (95% CI, 3.8 to 7.6 months) for platinum-based chemotherapy and PD-1/PD-L1 inhibitor in combination with chemotherapy, respectively.

### Overall Survival

**Figure 2.**
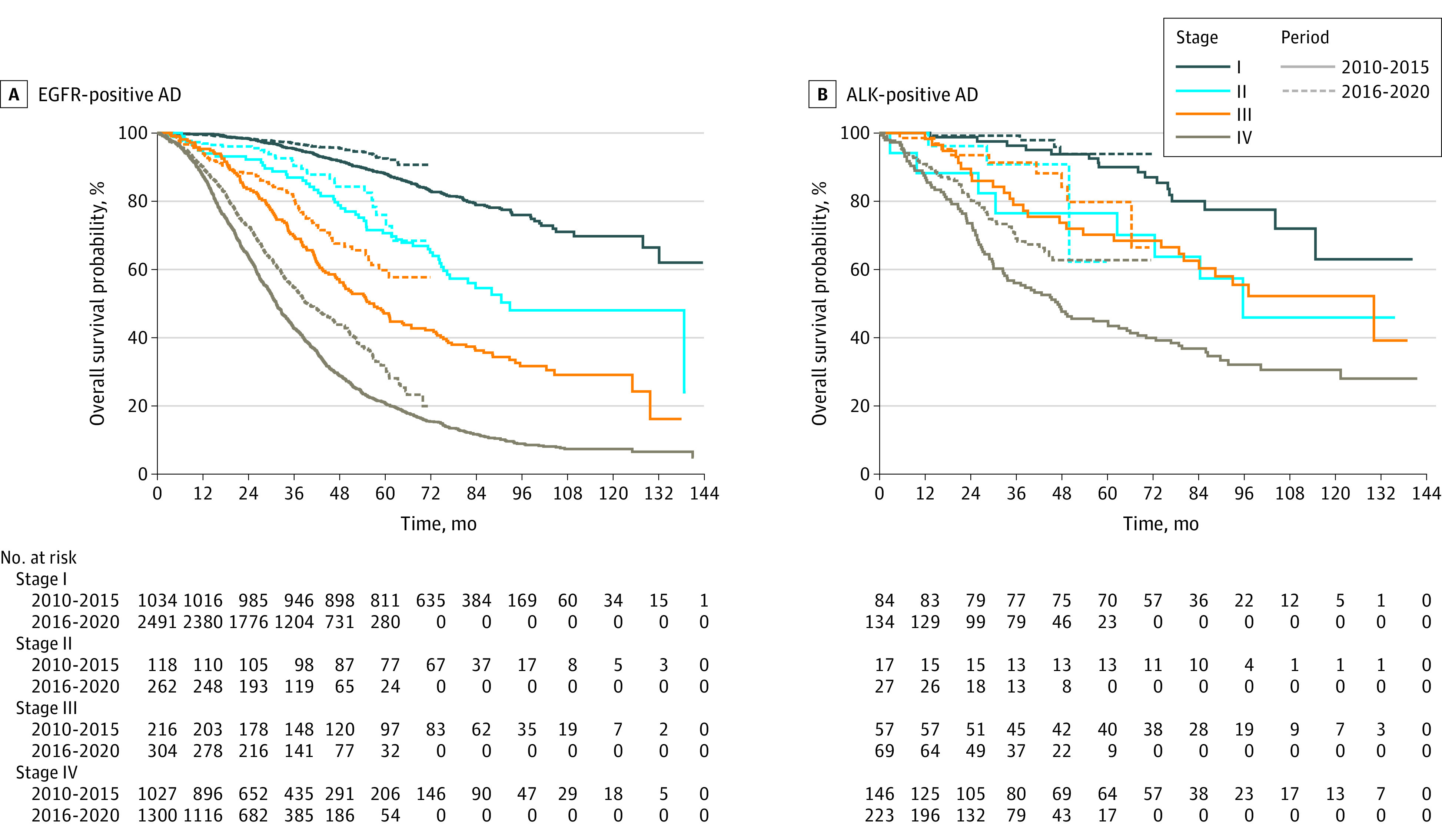
Overall Survival Curves For Patients With Druggable Variations and Adenocarcinoma (AD) A, Survival curves for patients with epidermal growth factor receptor (EGFR) variation are presented. B, Survival curves for patients with anaplastic lymphoma kinase (ALK) variation are presented.

**Figure 3.**
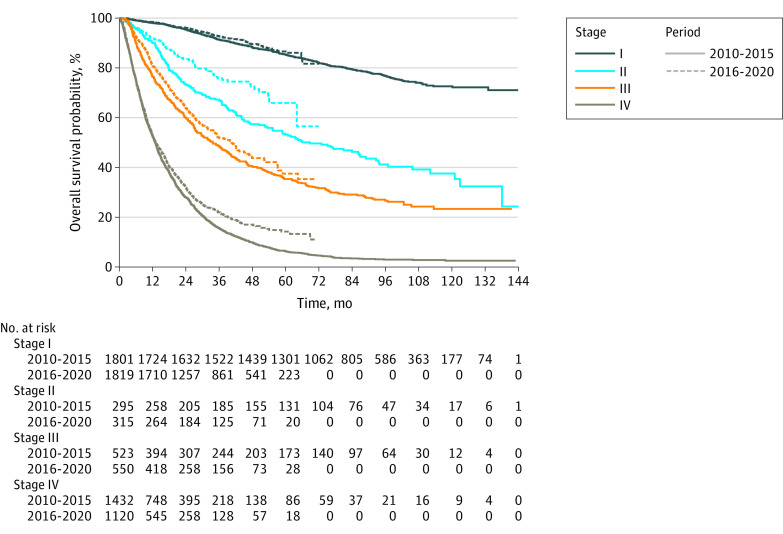
Overall Survival Curves for Patients With Adenocarcinoma (AD) and Without Druggable Variations

**Figure 4.**
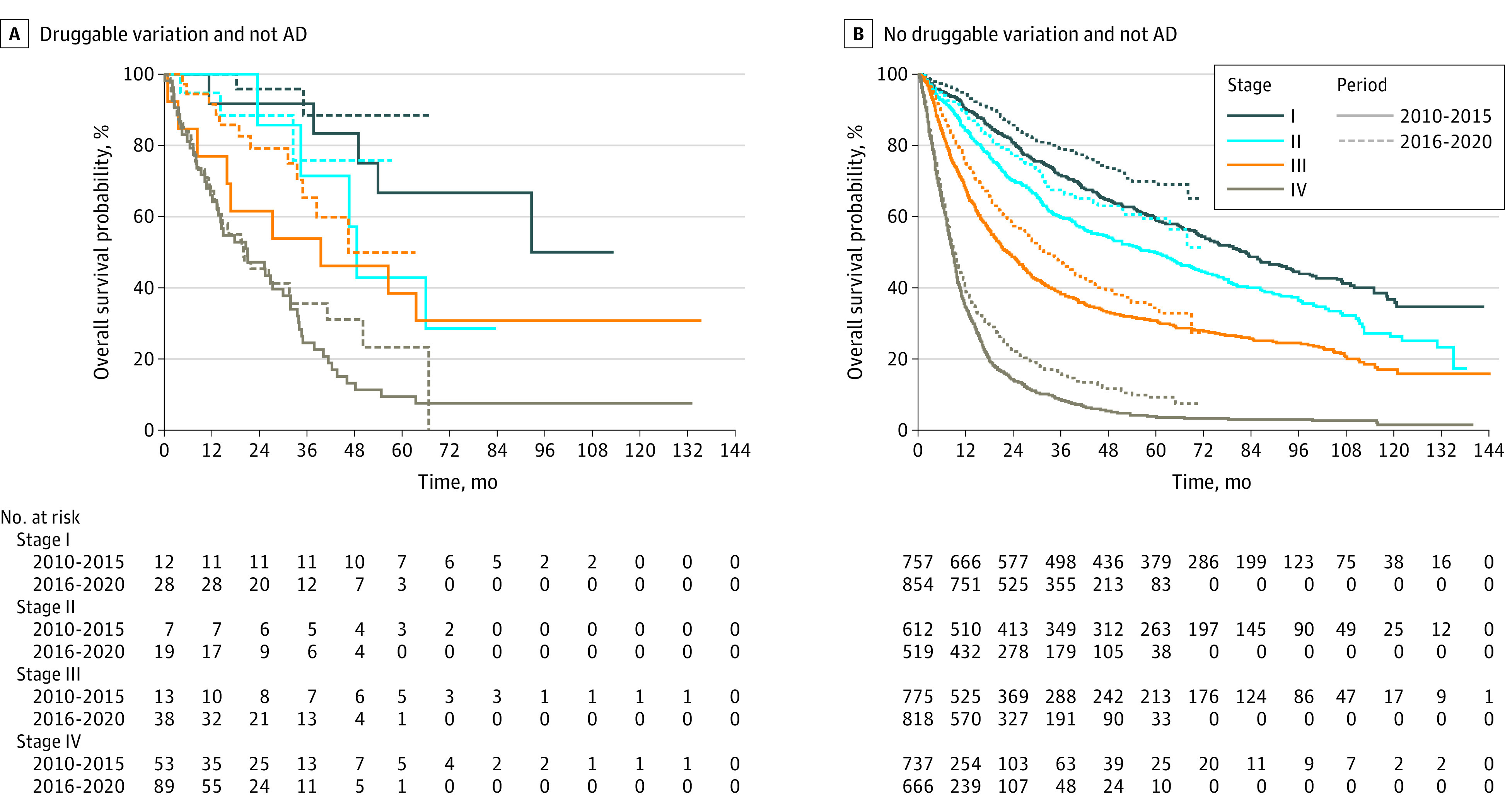
Overall Survival Curves for Patients Without Adenocarcinoma (AD) and With and Without Druggable Variations

The OS by clinical stage and major druggable variation is shown in Figure 2, Figure 3, and Figure 4 and eTable 2 in Supplement 1. In period I, the 3-year survival rates of patients with AD were 92.8% (95% CI, 91.8%-93.7%), 72.4% (95% CI, 68.3%-76.8%), 56.7% (95% CI, 53.4%-60.2%), and 28.7% (95% CI, 27.0%-30.4%) for stage I, II, III, and IV, respectively. The 3-year survival rates of patients with AD were 95.1% (95% CI, 94.4%-95.9%), 82.5% (95% CI, 79.1%-86.1%), 65.1% (95% CI, 61.8%-68.6%), and 42.4% (95% CI, 40.3%-44.7%) for each stage, respectively, in period II.

In patients with advanced-stage EGFR variation–positive AD who had received first-line EGFR-TKI, the median OS was 34.3 months (95% CI, 32.2 to 36.8 months) and 43.1 months (95% CI, 39.1 to 49.0 months) for period I (1087 patients) and II (1557 patients), respectively (*P* < .001). The 3-year survival rates of patients with advanced-stage EGFR variation–positive AD who had received first line EGFR-TKI were 48.0% (95% CI, 45.0%-51.1%) in period I and 57.0% (95% CI, 54.0%-60.1%) in period II (*P* < .0001). In period I, 1113 patients with EGFR variation–positive NSCLC received first- or second-generation EGFR-TKI as the first-line treatment. Among them, rebiopsy was performed in 781 patients (70.2%), and among these patients, the T790M variation rate was 353 patients (45.2%). In period II, 1528 patients with EGFR variation–positive NSCLC received first- or second-generation EGFR-TKI as the first-line treatment. Compared with period I, more efforts have been made to perform rebiopsy for the detection of resistance mechanisms and sequential treatment in period II. In period II, rebiopsy was performed in 1062 patients (70.2%), and the T790M variation rate among these patients was 464 patients (43.7%). More patients with T790M variation in period II (404 patients [87.1%]) received osimertinib compared with period I (265 patients [75.1%]).

The 3-year survival rate of patients with advanced-stage, ALK-positive AD who received first line ALK-TKI was 73.0% (95% CI, 63.0%-84.6%) in period I. It was 76.9% (95% CI, 70.3%-84.1%) in period II (*P* = .55).

In patients without AD, 3-year survival rates were 72.0% (95% CI, 68.8%-75.3%), 60.0% (95% CI, 56.2%-64.1%), 38.9% (95% CI, 35.6%-42.5%), and 9.7% (95% CI, 7.9%-12.1% for clinical stages I through IV in period I, respectively. In period II, the 3-year survival rates of patients without AD were 79.3% (95% CI, 76.3%-82.4%), 67.3% (95% CI, 62.8%-72.1%), 48.2% (95% CI, 44.5%-52.3%), and 18.1% (95% CI, 15.1%-21.6%) for clinical stages I through IV, respectively. In patients with clinical stage IV non-AD NSCLC with any major druggable variation, the 3-year survival rates were 24.5% (95% CI, 15.3%-39.3%) and 35.5% (95% CI, 25.0%-50.5%) in periods I and II, respectively (*P* = .21).

## Discussion

This cohort study found the trends in the epidemiology, treatment pattern, and outcomes in patients with NSCLC during the last 10 years in a clinical setting. The advantage of this study is that it enabled a comprehensive analysis of clinical, pathological, and molecular data in detail. It also has merits in showing how well-known advances in the diagnosis and treatment of NSCLC are being applied in clinics in a timely manner. In addition, this study’s results may identify unmet needs to improve the outcomes of NSCLC.

There was a difference in the incidence and mortality of NSCLC between periods I and II. As a recent trend in NSCLC, the proportion of never smokers and early-stage NSCLC increased in period II compared with period I. Ethnic differences in clinical behaviors of lung cancer have been found between Asian and White patients.^11^ Additionally, approximately 10% of patients with lung cancers were never smokers in the US, and more than 30% of patients with lung cancer were never-smokers^12,13^ The national smoking cessation program and national screening program for lung cancer have been actively implemented in the last 5 years. Accordingly, the Korean government enforced tobacco control policies and increased tobacco taxes in 2015.^14^ The high proportion of early clinical stage in lung cancer is also associated with the national lung cancer screening in South Korea.^15^ In 2016, the Korean Ministry of Health and Welfare announced the launch of the Korean Lung Cancer Screening project (K-LUCAS) in accordance with the guideline of lung cancer screening using low-dose computed tomography. Additionally, due to the high rate of performance of molecular tests, the proportion of patients with advanced NSCLC with druggable variations, including rare variations, has increased. Additionally, NGS has been increasingly implemented since it has been reimbursed in Korea in 2017.

An in-depth understanding of the evolution of molecular profiling and matched treatment achieved good clinical results. It is practically difficult to conduct all molecular studies considering the risk of biopsy, success rate, turnaround time, and cost-effectiveness. To date, our institute’s practice has been a sequential testing strategy in which EGFR PCR and ALK IHC have been reflex tests since 2013 in AD and non-AD groups, regardless of clinical stage. In cases of EGFR wild type and ALK-negative NSCLC, NGS is usually performed in patients who have archival tissue. PD-L1 testing for immunotherapy has been performed since 2018. Our institute has had a sequential testing strategy considering the high prevalence of EGFR variation–positive or ALK-positive NSCLC and the turnaround time of NGS. A recent opinion is that the up-front NGS test is a feasible and cost-effective method compared with sequential testing strategies in an EGFR variation–predominant population.^16^

The quest for more effective treatment strategies focuses on targeted therapies using comprehensive molecular tests and immunotherapy. Targeted therapies for EGFR variation, ALK fusion, *ROS1* fusion, *BRAF* V600E variation, and *NTRK1/2/3* gene fusion–positive NSCLC have been reimbursed. Recently, targeted therapies for *RET* rearrangement, *MET* exon 14 skipping, and *KRAS* G12C variation have been approved by the KFDA, and reimbursement is in progress. First-line immunotherapy with or without chemotherapy was reimbursed in patients based on a phase III study.^17,18,19^ In addition, adjuvant osimertinib for early-stage EGFR variation–positive NSCLC showed a survival benefit,^20^ and this treatment has been approved by the KFDA since 2021. In future investigations, we may be able to identify emerging driver sequence variations through NGS tests and describe in detail the outcomes of targeted therapy. In addition to ongoing efforts to find emerging driver sequence variations, there is a need for a deep understanding of the clinical setting, which includes factors before and after treatment and limitations of NGS implementation.

### Limitations

This retrospective cohort study has several limitations, including the known challenges of a retrospective, single-center study. Technical issues include deidentification, missing values, and biases, which can affect generalizability.

## Conclusions

Collectively, the results of this large-scale cohort study may suggest a paradigm shift in diagnosis, treatment, and outcomes over the past decade. The incidence of early-stage NSCLC and never-smokers increased. The OS of patients with all-stage NSCLC increased, with larger differences for stages III to IV. This study revealed the diversity of treatment practices and outcomes in NSCLC in a clinical setting. This cohort study may continue to serve as the foundation for the future development of large-scale, timely cohorts in routine clinical practice.

## Notes

Supplement 1. **eFigure 1.** Yearly Distribution of Clinical Stage **eFigure 2.** Recurrence-Free Survival for Patients With Up-front Surgery by Pathological Stage I–IIIA **eFigure 3.** Recurrence-Free Survival for Patients With Up-front Surgery by Sequence Variation Type **eFigure 4.** Sankey Diagram for Treatment Patterns in Patients With Clinical Stage IIIA and IIIB or C **eTable 1.** Distribution of Any Druggable Sequence Variation **eTable 2.** Survival Outcomes With Major Druggable Sequence Variations

Supplement 2.
Data Sharing Statement
