# Supplementary material for: Trends in Survival Rates of Non–Small Cell Lung Cancer With Use of Molecular Testing and Targeted Therapy in Korea, 2010-2020
Source: JAMA Netw Open. 2023 Mar 16;6(3):e232002. doi: 10.1001/jamanetworkopen.2023.2002 (PMC10020884; doi:10.1001/jamanetworkopen.2023.2002)
Supplement: Supplement 1. — eFigure 1. Yearly Distribution of Clinical Stage eFigure 2. Recurrence-Free Survival for Patients With Up-front Surgery by Pathological Stage I–IIIA eFigure 3. Recurrence-Free Survival for Patients With Up-front Surgery by Sequence Variation Type eFigure 4. Sankey Diagram for Treatment Patterns in Patients With Clinical Stage IIIA and IIIB or C eTable 1. Distribution of Any Druggable Sequence Variation eTable 2. Survival Outcomes With Major Druggable Sequence Variations [file jamanetwopen-e232002-s001.pdf]

Supplemental Online Content

Chi SA, Yu H, Choi YL, et al. Trends in survival rates of non–small cell lung cancer with use of molecular testing and targeted therapy in Korea, 2010-2020. *JAMA Netw Open.* 2023;6(3):e232002. doi:10.1001/jamanetworkopen.2023.2002

eFigure 1. Yearly Distribution of Clinical Stage

eFigure 2. Recurrence-Free Survival for Patients With Up-front Surgery by Pathological Stage I–IIIA

eFigure 3. Recurrence-Free Survival for Patients With Up-front Surgery by Sequence Variation Type

eFigure 4. Sankey Diagram for Treatment Patterns in Patients With Clinical Stage IIIA and IIIB or C

eTable 1. Distribution of Any Druggable Sequence Variation

eTable 2. Survival Outcomes With Major Druggable Sequence Variations

This supplemental material has been provided by the authors to give readers additional information about their work.

© 2023 Chi SA et al. *JAMA Network Open.*

eFigure 1. Yearly Distribution of Clinical Stage

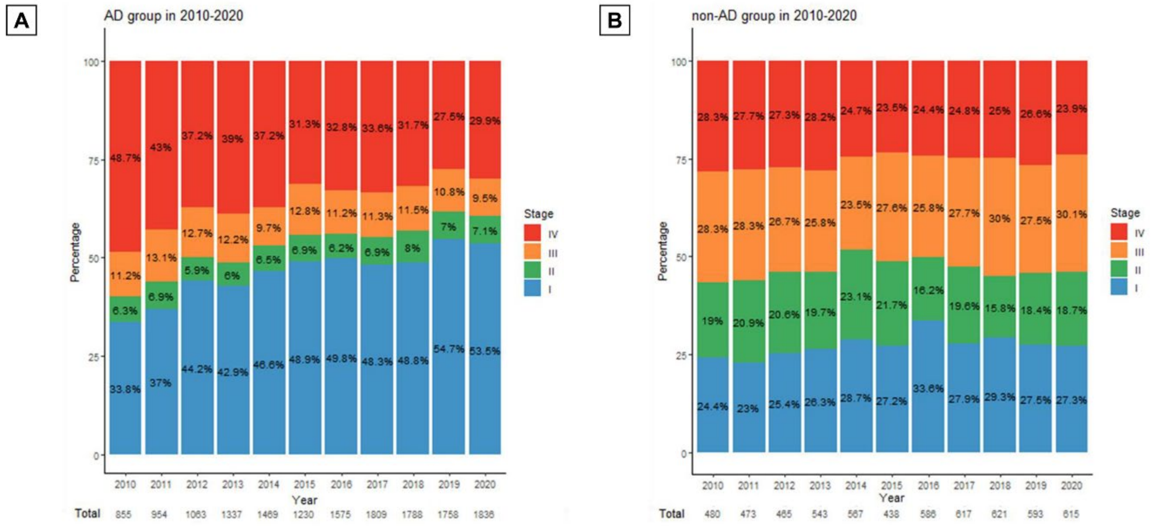

© 2023 Chi SA et al. *JAMA Network Open.*

eFigure 2. Recurrence-Free Survival for Patients With Up-front Surgery by Pathological Stage I–IIIA

For the AD group, (A), (B), and (C) represent RFS curves for those with *EGFR* variation-positive, *ALK* variation-positive, and *EGFR* and *ALK* wild-type NSCLC, respectively. (D) and (E) represent RFS curves for the non-AD group with and without any major druggable sequence variation, respectively.

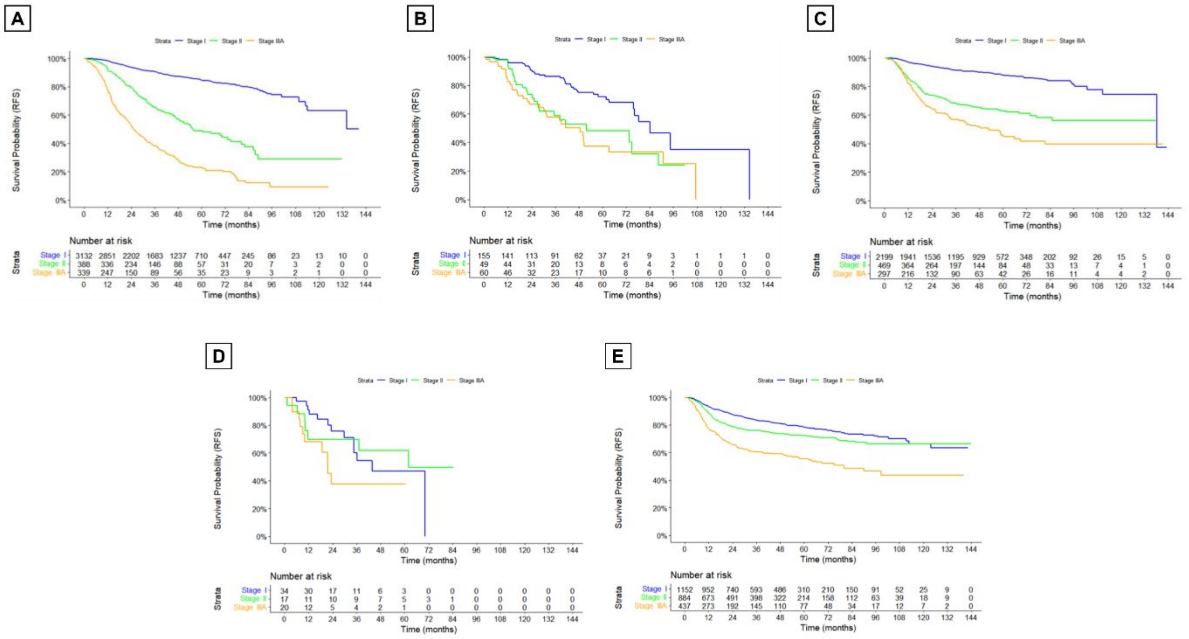

© 2023 Chi SA et al. *JAMA Network Open.*

eFigure 3. Recurrence-Free Survival for Patients With Up-front Surgery by Sequence Variation Type (A) Stage I (B) Stage II (C) Stage IIIA according to *EGFR* variation-positive, *ALK* variation-positive, and *EGFR* and *ALK* wild-type NSCLC.

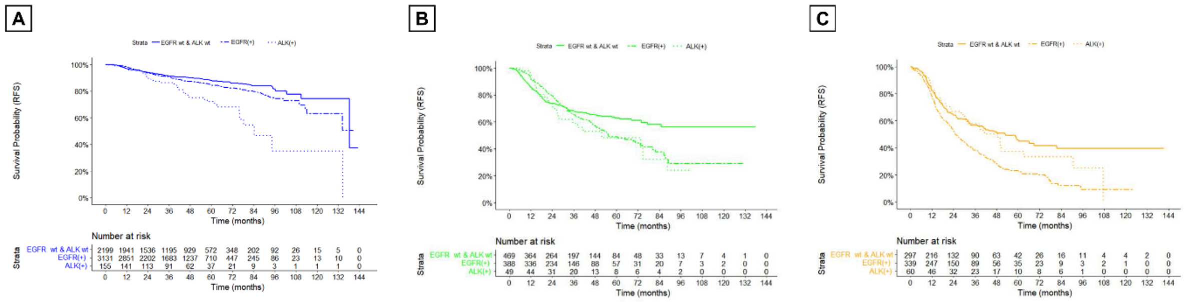

© 2023 Chi SA et al. *JAMA Network Open.*

eFigure 4. Sankey Diagram for Treatment Patterns in Patients With Clinical Stage IIIA and IIIB or C

The width of the arrows is proportional to data flow quantity. CCRT, concurrent chemo-radiotherapy; OP, operation; Tx, treatment; RT, radiotherapy; NED, no evidence of disease.

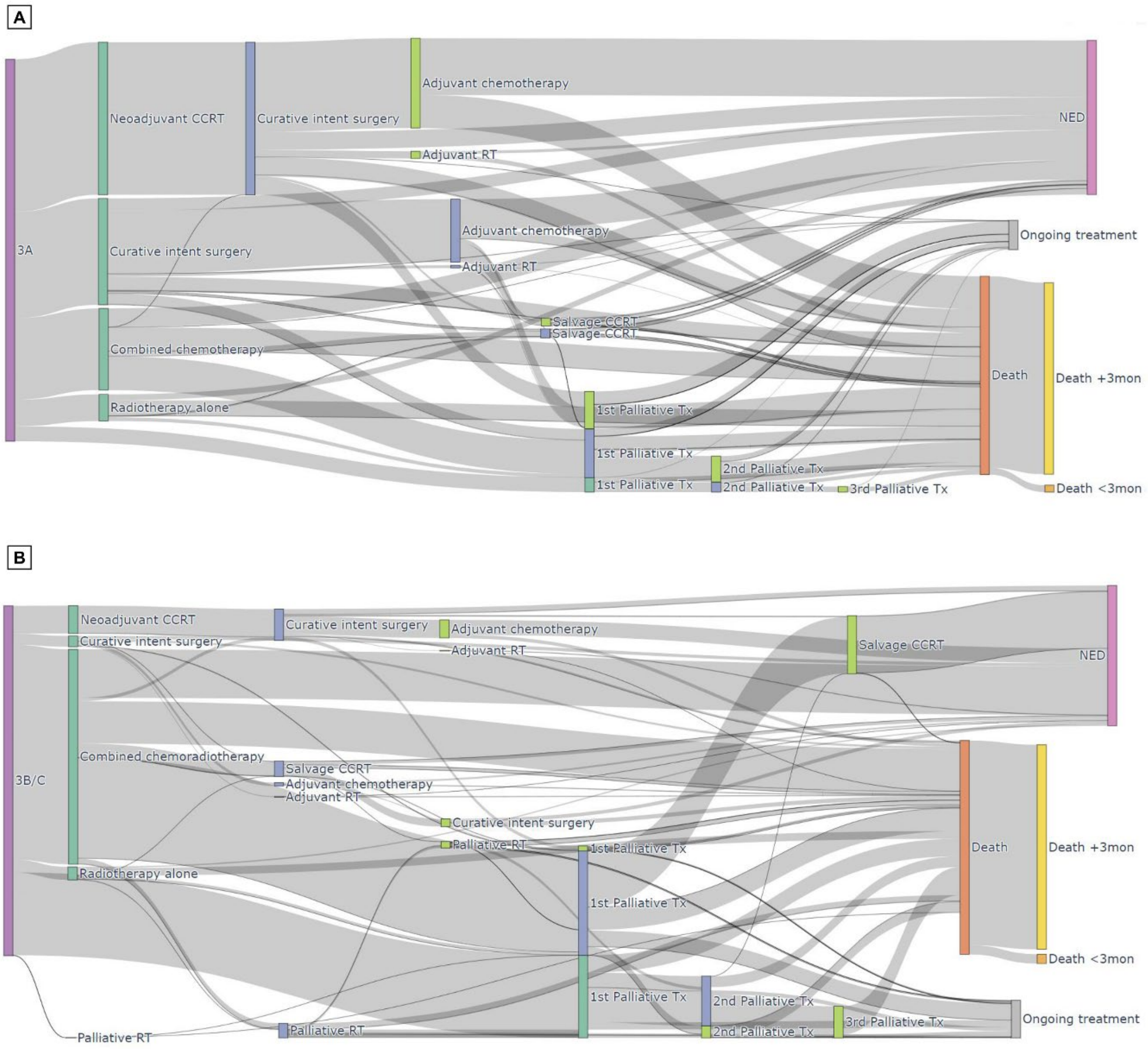

© 2023 Chi SA et al. *JAMA Network Open.*

eTable 1. Distribution of Any Druggable Sequence Variation

No. (%)	Overall (n = 21,978)	AD group (n = 15,925)				Non-AD group (n = 6,053)			
		Period I (n = 7,112)	Period II (n = 8,813)	SMD[d]	*P* value[c]	Period I (n = 2,998)	Period II (n = 3,055)	SMD[c]	*P* value[c]
Any major druggable variation[a]	8,122 (37.0)	2,877 (40.5)	4,985 (56.6)	0.67	<.001	86 (2.9)	174 (5.7)	0.86	<.001
EGFR variation	6,977 (31.7)	2,410 (33.9)	4,376 (49.7)			58 (1.9)	133 (4.4)		
ALK rearrangement	793 (3.6)	306 (4.3)	457 (5.2)			8 (0.3)	22 (0.7)		
ROS1 rearrangement	68 (0.3)	24 (0.3)	40 (0.5)			1 (0.0)	3 (0.1)		
RET rearrangement	69 (0.3)	23 (0.3)	41 (0.5)			3 (0.1)	2 (0.1)		
BRAF V600E variation	59(0.3)	28 (0.4)	22 (0.2)			4 (0.1)	5 (0.2)		
MET exon14 skipping	18 (0.1)	0 (0)	14 (0.2)			1 (0.0)	3 (0.1)		
KRAS G12C variation	136 (0.6)	85 (1.2)	34 (0.4)			11 (0.4)	6 (0.2)		
NTRK1/2/3 gene fusion positive	2 (0.0)	1 (0.0)	1 (0.0)			0 (0)	0 (0)		
Without any major druggable variation[b]	13,856 (63.0)	4,235 (59.5)	3,828 (43.4)			2,912 (97.1)	2,881 (94.3)		
Emerging druggable variations[c]	169 (0.8)	11 (0.2)	106 (1.2)			2 (0.1)	50 (1.6)		
No driver variation (NGS)	731 (3.3)	303 (4.3)	116 (1.3)			99 (3.3)	213 (7.0)		
EGFR (-) and *ALK* not rearranged	9,618 (43.8)	2,487 (35.0)	3,424 (38.9)			1,425 (47.5)	2,282 (74.7)		
Molecular test was not performed	3,338 (15.2)	1,434 (20.2)	182 (2.1)			1,386 (46.2)	336 (11.0)		

Abbreviations: NSCLC, non-small cell lung cancer; EGFR, epidermal growth factor receptor; ALK, anaplastic lymphoma kinase; ROS1, ROS proto-Oncogene 1; RET, rearranged during transfection; BRAF, V-Raf murine sarcoma viral oncogene homolog B; MET, mesenchymal epithelial transition; KRAS, Kirsten rat sarcoma viral oncogene homolog; NTRK, neurotrophic tyrosine receptor kinase; NGS, next-generation sequencing; SMD, standardized mean difference.

[a] Any druggable variation denotes those who had at least one of the major druggable variations, including *EGFR*, *ALK*, *ROS1*, *RET*, *MET exon 14 skipping*, *BRAF V600E*, *KRAS G12C*, and *NTRK*.

[b] Without any druggable variation denotes those who do not have any of the eight druggable variations. There are four types of variations without any druggable variations: emerging druggable variations and no driver variation (NGS) and EGFR (-) and ALK not rearranged, and molecular tests were not performed.
[c] Emerging druggable variations denotes those who had *ERBB2* or *MET* amplification.
[d] Chi-square test or SMD was used to compare the presence of any major druggable variation between the two periods.

© 2023 Chi SA et al. *JAMA Network Open.*

eTable 2. Survival Outcomes With Major Druggable Sequence Variations

**AD group (n = 15,673)**								
**Subgroup**	**Clinical stage**	**Period I (2010–2015)**			**Period II (2016–2020)**			**Log-rank *P* value**
		**N (Event, %)**	**Median OS (months)**	**3-year survival rate (%)**	**N (Event, %)**	**Median OS (months)**	**3-year survival rate (%)**	
AD (n = 15,673)	I	2,972 (20.4)	*Not reached*	92.8 (91.8–93.7)	4,476 (5.5)	*Not reached*	95.1 (94.4–95.9)	<.001
	II	443 (51.5)	81.8 (71.3–93.5)	72.4 (68.3–76.8)	617 (17.8)	*Not reached*	82.5 (79.1–86.1)	<.001
	III	819 (67.5)	43.4 (40.2–49.6)	56.7 (53.4–60.2)	949 (35.9)	57.2 (51.3–71.9)	65.1 (61.8–68.6)	<.05
	IV	2,674 (91.2)	20.6 (19.8–21.8)	28.7 (27.0–30.4)	2,723 (54.3)	28.4 (27.0–30.6)	42.4 (40.3–44.7)	<.001
EGFR (+)[a] (n = 6,752)	I	1,034 (19.5)	*Not reached*	95.4 (94.1–96.7)	2,491 (3.6)	*Not reached*	97.0 (96.2–97.8)	<.001
	II	118 (44.1)	92.8 (76.3–139.1)	87.0 (81.0–93.3)	262 (11.8)	*Not reached*	90.4 (86.2–94.8)	.31
	III	216 (66.2)	57.0 (48.0–66.9)	69.9 (64.0–76.4)	304 (25.0)	*Not reached*	80.9 (76.1–86.0)	.01
	IV	1,027 (89.3)	31.3 (29.9–33.0)	42.8 (39.9–45.9)	1,300 (44.0)	39.6 (37.3–44.1)	54.7 (51.6–58.0)	<.001
ALK (+)[b] (n = 757)	I	84 (20.2)	*Not reached*	96.3 (92.2–100.0)	134 (3.0)	*Not reached*	99.2 (97.6–100.0)	.40
	II	17 (47.1)	95.8 (72.5–135.8)	76.5 (58.8–99.5)	27 (14.8)	*Not reached*	90.8 (79.3–100.0)	.93
	III	57 (45.6)	130.3 (84.2–139.1)	79.0 (69.0–90.3)	69 (13.0)	*Not reached*	91.3 (84.3–99.0)	.31
	IV	146 (65.8)	47.0 (33.6–68.1)	56.0 (48.5–64.8)	223 (27.4)	*Not reached*	69.1 (62.3–76.6)	<.05
EGFR and *ALK* WT[c] (n = 5,845)	I	854 (27.6)	*Not reached*	88.2 (86.0–90.4)	1,614 (7.3)	*Not reached*	93.1 (91.7–94.6)	<.001
	II	192 (59.9)	55.4 (43.9–72.9)	65.9 (59.4–73.1)	290 (23.4)	*Not reached*	75.1 (69.6–81.1)	<.05
	III	391 (74.7)	32.2 (27.7–39.7)	47.0 (42.3–52.3)	485 (46.6)	38.7 (32.5–46.1)	51.2 (46.4–56.5)	.15
	IV	1,014 (95.5)	13.1 (12.1–14.0)	16.4 (14.3–18.9)	1,005 (73.8)	12.7 (11.8–14.2)	21.1 (18.4–24.2)	.04
Others[d] (n = 2,319)	I	1,000 (15.2)	*Not reached*	93.7 (92.1–95.2)	237 (13.9)	*Not reached*	86.9 (81.7–92.4)	<.001
	II	116 (45.7)	102.2 (80.9–144.1)	67.8 (59.6–77.0)	38 (18.4)	*Not reached*	76.8 (62.8–94.0)	.14
	III	155 (59.4)	44.5 (27.0–68.1)	54.5 (47.0–63.1)	91 (33.0)	55.2 (46.1–70.6)	64.0 (53.4–76.7)	.14
	IV	487 (94.3)	13.2 (11.1–15.0)	15.9 (13.0–19.6)	195 (53.3)	24.5 (18.9–35.7)	39.7 (32.1–48.9)	<.001

© 2023 Chi SA et al. *JAMA Network Open.*

non-AD group (n = 5,997)								
Subgroup	Clinical stage	Period I (2010–2015)			Period II (2016–2020)			Log-rank *P* value
		N (Event, %)	Median OS (months)	3-year survival rate (%)	N (Event, %)	Median OS (months)	3-year survival rate (%)	
non-AD (n = 5,997)	I	769 (49.3)	82.7 (73.6–92.9)	72.0 (68.8–75.3)	882 (20.0)	*Not reached*	79.3 (76.3–82.4)	<.001
	II	619 (60.4)	58.3 (48.6–68.6)	60.0 (56.2–64.1)	538 (28.8)	*Not reached*	67.3 (62.8–72.1)	<.05
	III	788 (75.3)	22.9 (19.9–26.5)	38.9 (35.6–42.5)	856 (48.0)	34.0 (29.9–38.6)	48.2 (44.5–52.3)	<.001
	IV	790 (96.2)	9.2 (8.5–9.8)	9.7 (7.9–12.1)	755 (75.2)	10.1 (9.0–11.2)	18.1 (15.1–21.6)	<.001
Any major druggable variation[e] (n = 259)	I	12 (41.7)	92.7 (53.9–113.4)	91.7 (77.3–100.0)	28 (7.1)	*Not reached*	88.5 (74.1–100.0)	.32
	II	7 (71.4)	48.6 (34.5–83.7)	71.4 (44.7–100.0)	19 (15.8)	*Not reached*	75.8 (53.5–100.0)	.43
	III	13 (69.2)	39.5 (15.8–135.5)	53.9 (32.6–89.1)	38 (31.6)	46.5 (35.0–63.4)	65.3 (49.3–86.5)	.36
	IV	53 (92.5)	21.0 (13.7–31.9)	24.5 (15.3–39.3)	89 (56.2)	20.1 (14.4–41.1)	35.5 (25.0–50.5)	.44
EGFR and *ALK* WT[c] (n = 3,688)	I	392 (46.4)	83.4 (75.6–110.0)	74.3 (70.0–78.9)	675 (19.6)	*Not reached*	80.5 (77.2–84.0)	.01
	II	323 (58.5)	61.7 (49.0–70.9)	61.8 (56.7–67.4)	441 (26.5)	*Not reached*	70.2 (65.3–75.3)	<.05
	III	384 (75.5)	23.7 (19.6–28.4)	39.2 (34.6–44.5)	646 (46.6)	34.9 (29.9–41.0)	48.7 (44.3–53.4)	<.05
	IV	316 (96.5)	9.2 (8.5–10.4)	9.6 (6.8–13.5)	511 (78.7)	9.6 (8.4–10.6)	15.6 (12.3–19.7)	<.05
Others[d] (n = 2,050)	I	365 (52.6)	73.7 (66.4–94.6)	68.8 (64.1–73.8)	179 (23.5)	*Not reached*	73.2 (66.2–81.0)	.15
	II	289 (62.3)	53.3 (40.1–75.8)	57.7 (52.2–63.8)	78 (44.9)	36.5 (30.6–71.6)	50.4 (39.0–65.1)	.35
	III	391 (75.2)	22.0 (17.9–26.7)	38.0 (33.5–43.2)	172 (57.0)	23.6 (17.9–36.6)	42.6 (35.2–51.7)	.66
	IV	421 (96.4)	8.4 (7.6–9.3)	7.9 (5.7–11.0)	155 (74.8)	8.2 (7.4–11.6)	17.0 (11.3–25.5)	.04

Abbreviations: OS, overall survival; NSCLC, non-small cell lung cancer; AD, adenocarcinoma; non-AD, non-adenocarcinoma; EGFR, epidermal growth factor receptor; ALK, anaplastic lymphoma kinase; WT, wild type.

Not reached indicates that the median OS cannot be calculated, and NA indicates that the upper 95% confidence interval of the median OS was not calculated.

[a] *EGFR*(+) denotes those with *EGFR* variation-positive NSCLC.

[b] *ALK*(+) denotes *ALK*-positive NSCLC.

[c] *EGFR* and *ALK* WT denote those with *EGFR* and *ALK* wild-type NSCLC, respectively.

[d] In the AD group, others denote those who did not belong to *EGFR* variation-positive, *ALK*-positive, or *EGFR* and *ALK* wild-type NSCLC. In the non-AD group, others denote those who do not have any key druggable variations or belong to those with wild-type *EGFR* and *ALK* NSCLC.

© 2023 Chi SA et al. *JAMA Network Open.*

[e] Any druggable variation denotes those who had at least one of the major druggable variations, including *EGFR*, *ALK*, *ROS1*, *RET*, *MET exon 14 skipping*, *BRAF V600E*, *KRAS G12C*, and *NTRK*.

© 2023 Chi SA et al. *JAMA Network Open.*
