# Supplementary material for: Trends in Survival Rates of Non–Small Cell Lung Cancer With Use of Molecular Testing and Targeted Therapy in Korea, 2010-2020
Source: JAMA Netw Open. 2023 Mar 16;6(3):e232002. doi: 10.1001/jamanetworkopen.2023.2002 (PMC10020884; doi:10.1001/jamanetworkopen.2023.2002)
Supplement: Supplement 2. — Data Sharing Statement [file jamanetwopen-e232002-s002.pdf]

Data Sharing Statement

Chi. Trends in Survival Rates of Non–Small Cell Lung Cancer With Use of Molecular Testing and Targeted Therapy in Korea, 2010-2020. *JAMA Netw Open*. Published online March 16, 2023. doi:10.1001/jamanetworkopen.2023.2002

Data
Data available: No

© 2023 Chi SA et al. *JAMA Network Open*.
